# Development of patient support devices for execution of clinical radiotherapy for cancer patients: A preliminary report

**DOI:** 10.4103/0971-6203.29195

**Published:** 2006

**Authors:** N. K. Babu, Bakshish Singh, S. Namrata, B. K. Mohanti, R. Ravichandran, K. E. Ghamrawy

**Affiliations:** Department of Radiotherapy, National Oncology Center, Royal Hospital, Muscat, Sultanate of Oman; * Department of Medical Physics Unit, National Oncology Center, Royal Hospital, Muscat, Sultanate of Oman

**Keywords:** Low-cost patient positioning devices, serial electronic portal imaging detector imaging, patient immobilization

## Abstract

The present paper illustrates our attempt to design and test the reproducibility of low-cost patient positioning devices prepared in-house in our radiotherapy department. Rigid thermocole boards with angulations, scales and support were designed as breast, pelvis and head positioning devices. Reproducibility and accuracy were tested by serial electronic portal imaging detector imaging.

The positioning devices (with or without superimposed moulds) showed variations within 2-3 mm on serial treatment days which were within acceptable limits.

It is therefore concluded that low-cost patient positioning devices for head, breast and pelvis (the common sites of treatments in radiotherapy) can be fabricated from available materials in-house. These have been shown to be resulting in accurate immobilization, can be customized for particular techniques and are considerably cheaper than commercially available solutions.

## Introduction

Success in radiotherapy is to deliver a high dose of radiation to the tumor tissue, at the same time keep the dose to surrounding normal tissues to the minimum, in order to reduce incidences of acute and late effects of ionizing radiation dose. Proper treatment planning and careful execution in the first course of radiation treatment is highly recommended, because patients with residual tumor or recurrence fall in the unfortunate category with markedly altered radio-sensitivity of the residual cancer as well as little further tolerance in the adjacent normal tissues. Therefore all the components of curative radiotherapy viz. selection of proper radiotherapy field, alignment of fields, beam direction, dose prescription, dose delivery and reproducibility in final execution should be accurately managed.

Earlier studies have shown that the tumor control probability (TCP) has a close bearing on the decision of radiation field placements. Significance of immobilization errors on the TCP have been studied in detail by applying theoretical models.[1] The effect of immobilization and localization errors were reported in head and neck cancer[2,3] and need for frequent verification films for detecting field placement errors was recommended.[4] From retrospective studies, they have shown 5 mm errors in 7-26% of the cases and above 10 mm in 6-23% of the set ups in various sites. The effect of different immobilization systems in radiation oncology have been reported for various sites.[5,6] The need for accurate positioning of the patient is a pre-requisite for successful radiotherapy.[7] Electronic portal imaging detector (EPID) verification system have become part of treatment machines. Using these systems we are able to study the reproducibility of execution of daily radiotherapy fractions.[8]

Patient information softwares address the daily variations in the electronic portal verification images against reproducible anatomical landmarks. Carefully thought out and developed treatment techniques employing field imaging, intelligent use of immobilization devices and well-trained and motivated therapy technologists are obvious crucial elements in the effort to reduce positioning errors. Multi-institutional studies addressing patterns of care in radiotherapy have clearly shown that there is no statistical significance of cure rates because of the type of treatment machines, but it is related to only the medical and technical skills available and physical support of treatment and other infrastructure.

Patient positioned in a normal comfortable posture with ability to remain in that treatment position for long duration forms the basic criteria, so that reproducibility during simulation and CT scanning could be achieved. Making decision on selection of multiple fields and beam direction of the fields will be possible when the radiation oncologist is satisfied with the selected posture of the patient.

Portal films are taken to confirm the execution accuracy either weekly once or twice. Conventionally in the X, Y, Z co-ordinate system, the table top is parallel to the XY plane, where the patient's longitudinal direction will be along the Y axis. Z plane refers to the depth of various structures and any variation in the Z axis on the treatment field has to be executed by the tilting of the collimator. Hard type supports without sagging effects are used to tilt the patient's axis either longitudinally or laterally. Head and neck supports are used for the Z axis tilts. After proper positioning of the patients in their expected treatment position, a thermoplastic cast is made to fix the required orientation. For the magnitudes of field sizes, their orientations with respect to couch lateral, longitudinal and transverse axes, IEC system is followed by the software to quantify field tilts and rotations both in the simulator and treatment machine.

Set up errors are defined as the difference between the actual and intended position of the part of the patient that is irradiated, with respect to the treatment beam(s) during treatment. Using EPID images, the errors are expressed against either a simulator image or with respect to a digitally reconstructed radiograph (DRR). Daily variations during inter-fractions are expressed against some reference landmarks of either anatomical structures or some radio-opaque markers. Large number of studies have been conducted to assess the criteria for good clinical practice in patient positioning.[9] A report from the American Association of Physicists in Medicine (AAPM 2001) has outlined the clinical use of electronic portal imaging and their evaluation.

Based on the experience of established radiotherapy centers, patient support devices are commercially available from various manufacturers. Though these will be able to reproduce standard treatments, there is a need for having in-house fabrication efforts to tailor to the particular patient requirements and also for achieving finer end results. Also standard positioning accessories are made up of plastic or wooden boards with little facility for maneuverability. The department of radiotherapy at this center has started functioning recently and we were in the process of procuring and establishing patient support aids for the type of patients encountered. We have therefore attempted to develop patient-positioning devices in house, for radiotherapy of breast, pelvis, and cranio-spinal areas, using hard thermocole sheets. Our preliminary experience in using these devices and reproducibility in treatment set-ups achieved are highlighted.

## Materials and Methods

### Specifications of thermocole sheets

The thermocole materials used for fabrication of patient support devices are Styrofoam sheets supplied by M/s Arply Medical Systems, France (high density type thermocole), which are normally used for making shield cut outs with ‘mantle cutting machine.’ The cost of 40×40×8 cm sheet is about US$ 6.00. These boards are quite stable and maintain position without sagging, for a distributed load of more than 80 kg. The density of this material is ρ=34 kg/m^3^ which is about 30 times less than that of water (ρ=1000 kg/m^3^) and therefore light to handle by the positioning technicians. Because of low density, these sheets do not provide appreciable scattering effect during irradiation; and also will have less scatter than that of carbon fiber breast boards, which are commercially available. Transmission measurements (with a field size of 20×20 cm) using ion chamber showed an attenuation factor of 0.98 for 12 cm thickness, which corresponds to μ=0.0017 cm^−1^ for 6 MV X-ray beam. During positioning, below each patient, a large size disposable tissue sheet is put for each radiotherapy treatment in the linac machines. Therefore, the in-house fabricated sheets do not pose problems of cleanliness, as they do not get soiled. Sometimes a plastic sheet can cover the board to avoid direct contact to the skin of patients. We had two sets of each size of positioning boards, one for the linac rooms and the other for the simulator. A total number of three sets will suffice to use for a period of about 2 years.

### Analysis of field position variations in portal vision images

The positioning devices developed in this study have been used for patients treated in the high energy linear accelerator Clinac 2300 CD (M/s Varian AG, USA). EPID imaging was routinely carried out using Portal Vision Amorphous Silicon Flat Panel Detector, model aS 500 (M/s Varian AG, USA). This has maximum lateral travel range of 31.8 cm, longitudinal travel range +18.6 cm to −13.2 cm. For each view of radiotherapy field, a reference image must be set up first. The central axis must be aligned to the simulation field. The anatomy layer must be defined for at least three anatomical contours. The planned field edge/field aperture must be generated or drawn manually. The portal image acquired and to be reviewed must be displayed and compared to the reference image. Deviations are indicated on the image monitor. The noted variations for each view is averaged over number of images and standard deviations are expressed.

## Details of fabrication of devices

### Breast board

#### Basic requirements.

Breast boards support the patient's head with a tilt to the opposite ipsilateral side, with arm supported at the shoulder level. With post-operative patients, the resting arm is at an angle, along with support at the palm level. Angulated breast boards are needed for minimizing collimator tilts, which result in underdosing at the junction of tangential chest and anterior supra clavicle fields. Better coverage of internal mammary chain and achieving better dose distribution are other advantages. The angle of collimation may differ from patient to patient which should be achieved by slopes in supporting board under the patient's back.

#### Design and fabrication.

**Figure 1:**
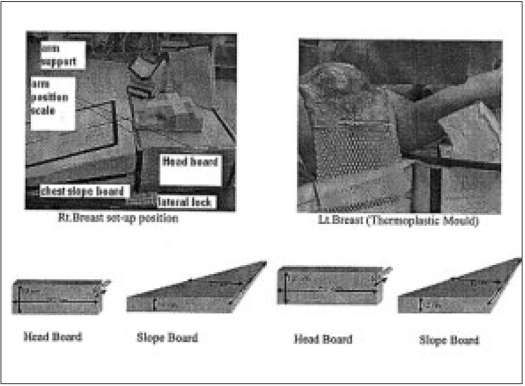
Technical details of the breast board developed using thermocole sheets

To meet the above needs two types of angulated breast boards (10° and 15°) are fabricated with the low density thermocole material. This has additional arm positioning support (re-producible scale coordinate system), along with thermoplastic mask fixation and planning-CT compatibility. Right breast and left breast treatment position can be achieved by a mobile platform. This also has a headboard, head rest, and arm supporter, all are detachable pieces with proper fixation. The method of fixation is designed with a plastic syringe hole insert and the syringe plunger fixed on to moving support. Using the above design, we can achieve variable angulations and longitudinal graduation. Therefore, reproducibility in the positioning of patient is achievable both at simulator and on the treatment table. Figure 1 shows the breast board with both angulations and indicating dimensions.

### Application on the patients

**Figure 2:**
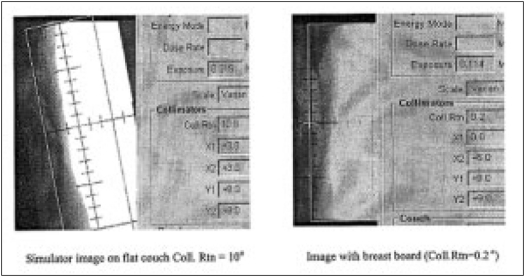
Comparison of simulator images of the same patient with the use of breast board

Patient set up can be seen with flat couch and with the breast board on simulator. Figure 2 compares the effect of achieving correct medial tangential projection with proper collimation. The angulations of breast board is variable e.g., (±5o). Arm positions are noted with arm positioning coordinate scale, longitudinal scale (y-coordinate) and horizontal scale (x-coordinate). With this method, reproducibility in arm rotation and position can be achieved.

### Prone-pelvic board

#### Basic requirements.

For the treatment of ca.rectum, for the pelvic fields, lateral portals should be used for a portion of treatment to avoid as much small bowel as possible. It is recommended that bladder distention and prone position are useful techniques for displacing the small bowel out of the pelvis.[10] Bulky abdomen and the presence of colostomy bag give lot of discomfort in this position on the flat couch and reproducibility is not very satisfactory because of intestines. Belly boards are used sometimes. We designed a prone pelvic board for the treatment of ca.rectum for the similar objective. Reproducibility is always better, while positioning the patient with respect to hard structures (bones). For prone set up, the whole abdomen intestines are hanging downwards by supporting pelvic region with lateral femur bone resting on the curved pelvic rest made of softened thermocole. By giving thigh rest, toe rest, and chest support, the patient can be positioned with good comfort. For daily set up, the patient has to be first aligned with respect to the pelvic board (by matching fixed markers on pelvic rest and thigh rest to patients skin markers using laser), then setting the patient in the machine for treatment.

#### Design and fabrication.

**Figure 3:**
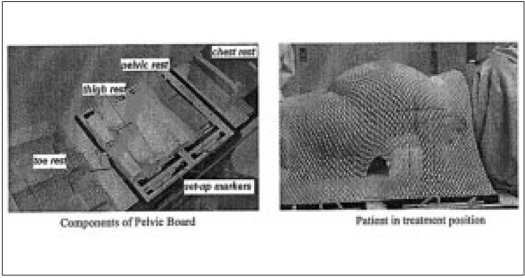
The pelvic board kept on the treatment table and its application for patient set up

The pelvic board is made up of low-density thermocole and designed with pelvic thermoplastic fixing for treatment planning, with CT compatibility. This device is designed for optimal treatment technique for Ca.rectum (one posterior and two lateral beams). As thermocole material has no significant effect on beam quality (density as close to air), this can be used for under couch treatment also (for ca.cervix, prostate, bladder treatments). Figure 3 indicates the technical details of the components of pelvic board.

### Supine-pelvic board

#### Basic requirements.

In the treatment of bulky patients, the reproducibility is a problem. Particularly treating multi-fields with isocentric technique (3D-conformal radiotherapy), the set up error makes a lot of variation on dose delivery. Also it is normally experienced that bulky patient with pelvic thermoplastic mould having worse reproducibility compared to without mould fixation because of development of folds. Also the internal organ movement is more with pelvic mould system. Mostly set up errors for bulky patients are due to large soft gluteus (back) muscles resting on the couch.

#### Design and fabrication.

With these requirements, we have designed a simple device supine pelvic board with thermocole material along with a knee rest. The thickness of thermocole are 3 and 5 cm with lower edge sloped, such that the curvature of gluteus muscle is just touching along the thermocole slope. The thickness of the thermocole varies as per the patient's back contour requirements. Both the legs resting on the knees support mount, thereby providing flat back properly resting and patient feels comfortable during treatment execution.

### Prone position face rest

#### Basic requirements.

For the treatment of cranio-spinal axis irradiation we need special care for positioning the patient for the cranial port with MLCs. Small change in the positioning could irradiate eyes and the same time missing some volume, which is to be treated.

#### Design and fabrication.

**Figure 4:**
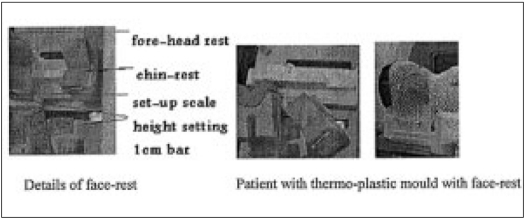
The various components of fore-head rest used in cranio-spinal treatments

We have fabricated cranial face rest with slope thermocole rest for the chest. For providing good comfort to paediatric age group of patients, we have used soft material such as thin sponge, fixed to the thermocole material. The chin and fore-head angle can be adjusted by 1 cm bar to best match to the patients. Figure 4 illustrates the design features achieved for the treatment of children.

## Results

### Breast board

**Figure 5:**
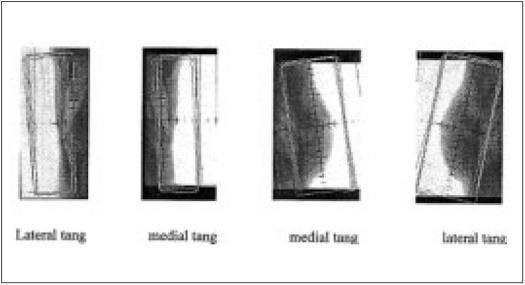
The beam edge plots uptained by epid in breast treatment on different days

Using the breast board fabricated in the department, so far we have treated 42 patients with 995 fractions and 1990 of medial and lateral tangential fields. Re-producibilty study carried in randomly selected five patients, using anatomical matching method (VARiS) in 25 medial and 25 lateral EPID images, revealed that the extent of variations were within acceptable results [Table 1 S. No. 1]. These are inter-patient variations on all the images taken together. The summary of beam edge plots obtained from sequential EPID study in these cases are represented in Figure 5.

**Table 1:** Observed shifts in treatment portals with electronic portal imaging detector verifications

*S. No.*	*Field description and variations*	*Total images*	*Treatment field*	*Mean variation in X coordinate mm*	*Mean variation in Y coordinate mm*	*Mean rotation mm*
1	Breast, variations cranio-caudal and lateral fields	25	Medial tang	2.2 ± 1.3	1.2 ± 0.8	1.2 ± 1.5
		25	Lateral tang	3.2 ± 3.0	1.0 ± 1.0	0.9 ± 1.0
2	Treatment with prone pelvic board and thermo plastic immobln.	5	Posterior Field	2.2	3	0
3	Prone Treatment with thermo-plastic mould alone	6	Posterior	3	5.5	0
		6	Lateral	8	4	1.5
4	Treatment with supine pelvic board	17	Anterior	2.1 ± 1.7	1.2 ± 2.8	0
		17	Lateral	1.3 ± 2.7	1.9 ± 2.3	0
5	Treatment with thermoplastic mould alone	6	Anterior	2.9 ± 1.8	5.7 ± 5.2	0
		6	Lateral	4.5 ± 3.5	3.4 ± 2.0	2.7 ± 2.6
6	Treatments for paediatric patients	13	Rt.Lateral	2.2 ± 1.6	3.2 ± 3.0	1.2 ± 1.3
		13	Lt.Lateral	1.8 ± 1.3	2.5 ± 2.6	0.7 ± 0.9

### Prone pelvic board

**Figure 6:**
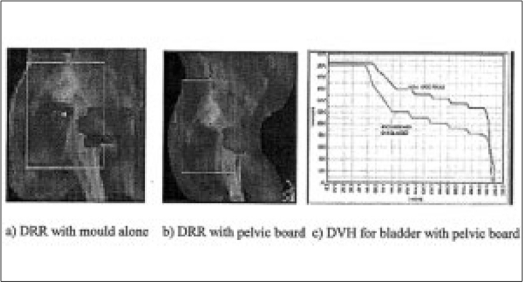
The effect of prone pelvic board in achieving less dose to bladder and small intestine

We have treated one patient with this device. Figure 6 gives the details of DRRs with orfit mould alone, pelvic board and orfit mould together and DVH for bladder with these two different positioning set up. The results on reproducibilty verification is shown by EPID comparing with simulation image [Table 1 S. No. 2]. Verification data for the same patient with normal treatment prone position (with thermoplastic mould alone) is shown in Table 1, S.no.3. The effect of using this board for achieving better treatment plan is seen in Figure 6 from the DRRs.

### Supine pelvic board

**Figure 7:**
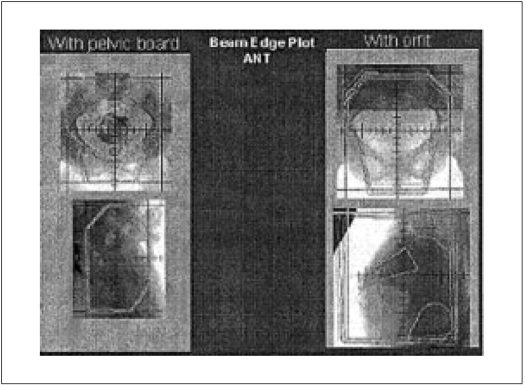
Beam edge plots uptained with pelvic board and orfit and orfit alone

We have treated two bulky patients with this device. The comparison of the reproducibilty with the pelvic board and with normal thermoplastic mould alone are shown in Table 1, S. no. 4 and 5. The comparison of EPID beam edge plots with and without pelvic board is shown in Figure 7.

### Prone position face rest

**Figure 8:**
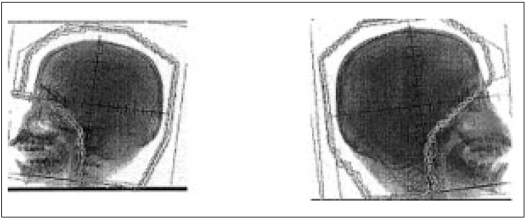
Analysis of right and left lateral brain beam edge plots in a pediatric patients

With this device we have treated four patients with good results. Table 1, S.no. 6 highlights the data on mean variation in reproducibility of treatment executions in a group of EPID sequential images. Figure 8 shows the comparison of EPID images on different fractions of dose delivery.

## Discussion

The aim of this work is to highlight the requirement for in house fabrication of patient support material depending on individual patient's radiotherapy plan. The department is in the process of acquiring commercially available treatment aids. As a preliminary study we had assessed the efficacy of the indigenously fabricated patient support devices, all of them were made out of easily available thermocole material. These fabricated accessories have good mechanical strength and provide good reproducibility and patient comfort as that of commercially designed boards of similar specifications, at less cost.

There are two different aspects in the problem of immobilization. One is to properly maintain patient's outer contour during treatment, with respect to isocenter. Other is related to variation of internal structures during daily treatments. We do not have much control on the latter, because they arise due to involuntary movements of internal organs. The former aspect is taken care of by using Orfit type commercially available thermoplastic mould fixable to the treatment couch. This paper has described the supportive devices on which patient is positioned to maintain good comfort and to obtain reproducibility during positioning. For general treatments, on account of the workload on the machines, the recommended frequency of routine port films can be considered as one per week. Therefore, we will have 5-6 images for a period of 5-6 weeks radiotherapy. The shift in field co-ordinates in this report will refer to the mean of the variations observed from all portal images taken from many patients. The objective of the study is to validate that whether the in house fabricated devices are able to provide comparable results similar to already available methods in many institutions. It is emphasized that the devices described here do not take away the purpose of thermoplastic immobilization requirement, but add to comfort to patient support below the patient. However, the thermoplastic casts will be continuing to remain for fixing the patient in planned posture. We have mostly compared the results available from some studies from reviewed literature.[11] There are many recent reports on patient immobilization found in literature. But they mostly relate to specialized treatments like intensity modulated radiotherapy or stereotactic radiotherapy where the reproducibility requirements are much crucial than the type of treatments highlighted in this work. Our presented material will represent situations as that of large fields conventional radiation treatments. Goal of EPID verification is to minimize the probability of patients being treated with a large systematic error.[12] Various studies have indicated that about 10 images per patient are necessary to reduce set up errors. This paper has concentrated to stress the point about application of patient positioning devices to offer optimal radiotherapy treatments, rather than the role of more number of EPID images to precisely express the extent of standard deviations or quantify magnitude of errors. We indicated that this is preliminary report, for reference at a later date when we have other type of patients supportive devices included in the future and also to stress the availability of QA program to monitor the patient position during radiotherapy.

The cancer hospitals in developing countries have financial constraints and find difficulty to meet the increasing demands for purchasing innumerable patient support aids. Thermoplastic moulds have become routine immobilization method adopted globally. These moulds along with this simple, light weight thermocole boards could be solution for small centers who can deliver accurate radiotherapy even with less expensive isotope machines.

Our results show that reproducibility within 2-3 mm mean deviation for breast and other sites is easily achievable. Verhey *et al*.[11] have compared the immobilization capabilities of different techniques. For breast treatments, they have given a shift of 3 mm achievable with present day immobilization methods. In other sites also our results show variations within acceptable limits. We have made an attempt to illustrate the point that by using appropriate supports patient comfort could be increased, thereby facilitating better reproducibility over the 5-6 weeks external beam radiotherapy. With bigger abdomen and pelvic sizes, the use of better positioning boards minimize the hanging of abdomen downwards, intestines and bladder falling away from right and left lateral beams could be avoided. The dose to these normal structures could be minimized when comparing to other treatment positions. For pelvis treatment, we have outlined the need for additional patient support inside the thermoplastic mould to reduce the reproducibility errors in multiple fractions. Male genital organs (penis, scrotum) falling down, the exit dose from posterior beam is minimized with appropriate supports. We propose to continue the study in large number of patients.
